# Microbial Growth Curve Framework Provides Insights for Controlling Opportunistic Pathogens in Building Plumbing

**DOI:** 10.1021/acsestwater.5c01431

**Published:** 2026-04-10

**Authors:** Tolulope O. Odimayomi, Amy Pruden, Marc A. Edwards

**Affiliations:** Via Department of Civil and Environmental Engineering, 1757 Virginia Tech, Blacksburg, Virginia 24061, United States

**Keywords:** water age, *Legionella pneumophila*, growth curve, drinking water, building plumbing

## Abstract

The perception that high water retention time (WRT) in buildings increases microbial and pathogen growth drives costly flushing interventions, which lack a scientific framework to guide effective implementation. Here, we evaluate the effect of WRT from 1 to 21 days in an at-scale building plumbing rig with influent chloramine residuals of <0.2–2.5 mg/L as Cl_2_ and water heater set points of 40 and 60 °C. We found that the classic microbial growth curve consisting of lag, exponential growth, stationary, and decay phases provided a robust explanation of trends in bulk water total cell counts (TCC) and *Legionella pneumophila* over a wide range of conditions. We extended this observation to develop a framework to understand how various controls and operating conditions act to stop (e.g., cold temperatures, disinfectant) or reset (e.g., pasteurization in water heaters killing microbes and recycling nutrients) the growth curve as a function of WRT. Bulk water TCC and *L. pneumophila* reached a consistent peak/plateau at a building WRT of ∼7 days before decaying up to 90% at higher WRT. These findings suggest that recent guidelines recommending weekly flushing of buildings may sometimes be counterproductive and that very high WRT does not necessarily indicate microbial risk.

## Graphical abstract

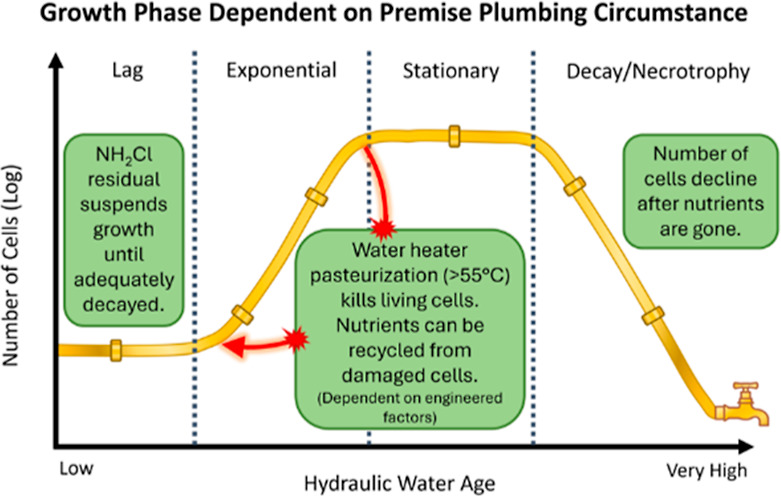

## Introduction

Contrary to steady improvements in drinking water treatment and associated public health outcomes over the past two centuries,^1,2^ problems with growth of opportunistic pathogens (OPs) in building plumbing are increasing. *Legionella pneumophila*, the OP responsible for Legionnaires’ disease, is linked to 93% of biofilm-related waterborne disease outbreaks and a majority of deaths, costing $2.4 billion annually in hospital care in the U.S.^3–5^ In response to such concerns it has become commonplace to recommend regular water flushing to reduce disease risks.^6–8^

The concept of water age was developed to explain certain aesthetic and public health problems for drinking water associated with its journey from the treatment plant to consumer taps.^9^ Conventionally, water age is a linear function of time, starting with “birthing” of water as it leaves the treatment plant and ending after it travels through the drinking water distribution system (DWDS) and emerges from a tap.^10–12^ Water retention time (WRT) further refers to the time the water specifically spends within the DWDS or building plumbing dependent on volume and flow rate (i.e., hydraulic water age). It is usually assumed that all aspects of microbial growth worsen as hydraulic water age increases in DWDSs and building plumbing (Figure 1a).^11,13,14^ Consequently, during the Covid-19 pandemic, consumers and building managers were instructed to routinely flush plumbing to decrease hydraulic water age, even though there was admittedly no evidence that longer stagnation increases disease risks from *Legionella* or other OPs.^15^

**Figure 1:**
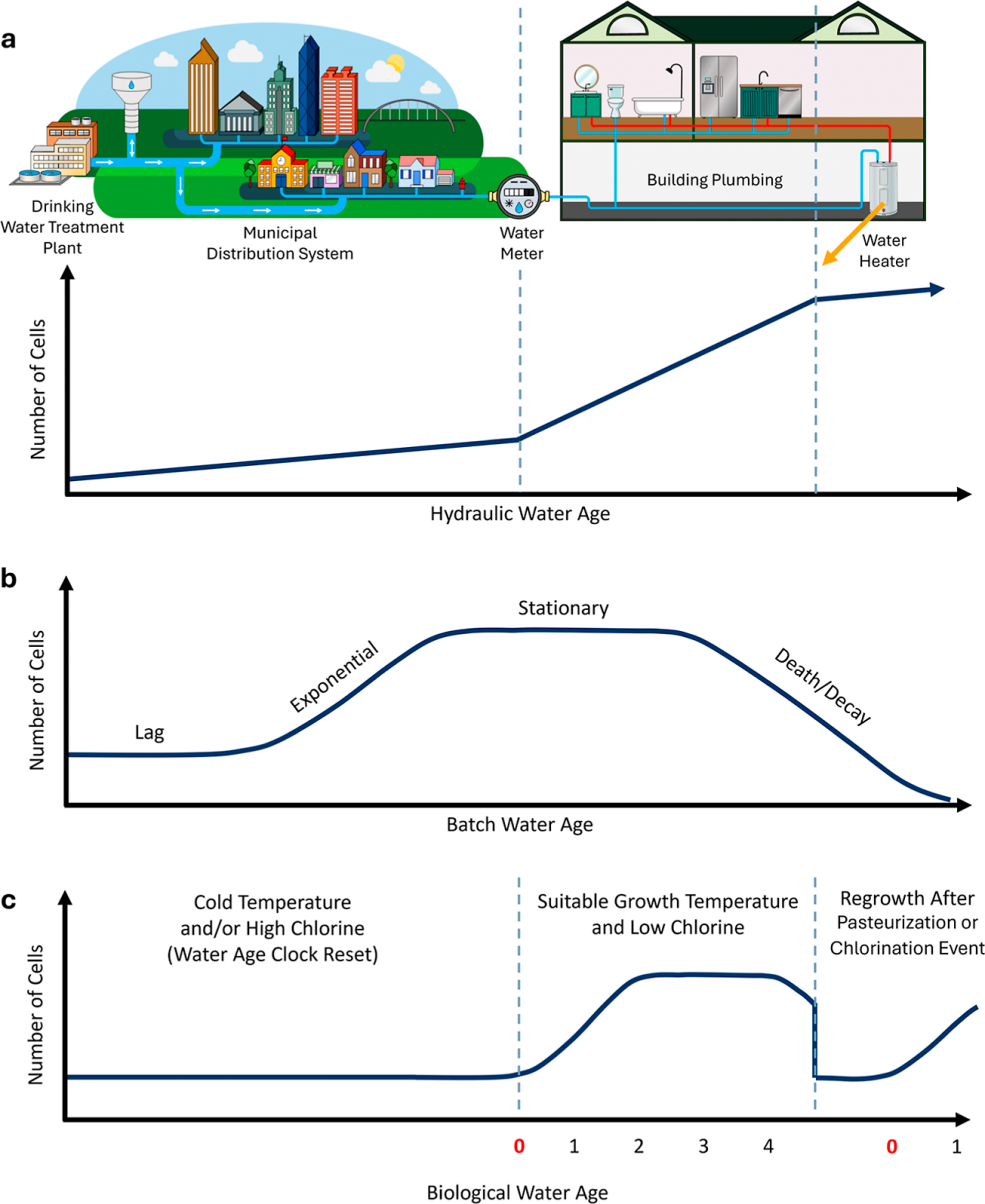
Conceptually aligning the classic microbial growth curve with trends in microbial growth along a drinking water distribution system (DWDS) from treatment to tap. (a) the conventional wisdom on hydraulic water age and microbial growth in a DWDS and associated building plumbing assumes unlimited growth, (b) the classic microbial growth curve for bacterial growth in a closed batch system, and (c) proposed biological water age framework for guiding opportunistic pathogen (OP) control in premise plumbing that accounts for situations that arrest growth or that reset the water age clock.

The U.S. Environmental Protection Agency (EPA) Surface Water Treatment Rule requires that a detectable disinfectant residual be maintained throughout the public DWDS to limit the potential for pathogen growth.^16^ As water travels through the DWDS, the disinfectant residual decays and oligotrophic bacteria grow.^17,18^ Even if the amount of disinfectant delivered to the building point-of-entry remains elevated, the small diameter pipes with high surface-area-to-volume ratios,^19–21^ long pipe lengths, low flow rates, low water use, warm temperatures, and high WRT in building plumbing can further diminish residual levels and provide the opportunity for pathogen colonization and growth.^22–25^

It is well-known that in a closed system or batch culture, where an organic carbon source is limited and no waste is removed, bacteria will grow in a predictable four phase pattern: lag, exponential, stationary, and death/decay.^26^ However, conventional wisdom regarding the effect of water age in DWDSs essentially assumes infinite exponential regrowth, ignoring the realities of the stationary and death phases triggered by the depletion of organic carbon, electron donors, and other nutrients (Figure 1a versus b). Regrowth refers to a loss of biological stability that leads to recovery of bacterial counts after disinfection.^27–30^ Here, we hypothesize that the classic microbial growth curve may provide a robust framework for understanding and predicting trends in microbial growth in drinking water as it is transported from the treatment plant to the tap. This approach represents the first integrated framework that simultaneously accounts for nutrient limitations, microbial decay, and biological water age reset events that can affect microbial growth trends before water reaches the tap. Furthermore, we extend this model to inform engineering strategies for the design, control, and operation of DWDS and building plumbing systems. For example, the microbial growth (i.e., biological water age) clock can conceptually be slowed or stopped at cold temperatures or at high chloramine disinfectant levels in water mains. On the other hand, the clock could be essentially reset after microbial death and associated nutrient recycling via necrotrophic growth or biomass decay, as can occur in hot water heaters at >60 °C (Figure 1c).^27,30^ While several countries have established regulations and guidelines to chill or heat water supplies to discourage pathogen growth,^3,31^ in warm climates or within buildings, the biological water age clock will continue to tick at temperatures that can promote OP growth and when disinfectant is low enough to allow growth.^32–36^

The aim of this study was to determine the extent to which the classic microbial growth curve describes observed patterns in total cell counts (TCC) and *L. pneumophila* abundance in a representative at-scale building plumbing rig. The rig was plumbed to the town water supply with nonrecirculating hot and cold-water lines, heating the hot water lines with a conventional electric tank water heater. The plumbing rig enabled systematic examination of how building WRT and other plumbing design and operation conditions, including hydraulics, filtration, and temperature, can influence disinfectant efficacy in controlling OPs, or dictate the dominant microbial growth phase. Furthermore, we were able to examine interactive effects of disinfectant level at the building point-of-entry with building WRT and these related factors. The findings inform the establishment of the microbial growth curve as a framework for understanding and predicting OP growth in premise plumbing as a function of biological water age and informing effective engineering design and controls to mitigate microbial risk.

## Methods

### Pipe Rig Design and Operation

The at-scale building plumbing rig is connected to the Town of Blacksburg, VA, chloraminated municipal water supply and was first commissioned in 2018 (Figure S1).^37^ As summarized in Busch et al.,^38^ before reaching the rig, town water is plumbed to flow through, or bypass, a set of granular activated carbon (GAC) filters (Pentair, Golden Valley, MN) capable of reducing total chlorine from >2.5 mg/L to 0.05 mg/L as Cl_2_. The water that entered the rig (influent), was a blend of the untreated and GAC-filtered town water. The proportion of GAC-bypassed town water was adjusted from 0% to 100% to achieve the desired influent chloramine level. Influent water flowed to a manifold that supplied 8 cold and 8 hot cross-linked polyethylene (PEX-B) water pipes (SharkBite, Atlanta, GA); each of which was 40.84 m long. Flow was either distributed directly to cold-water experimental conditions or into a 72 L (19-gallon) electric water heater before distribution to hot-water pipes. Water heater set points of 40 and 60 °C were selected to represent temperatures that are near the low and high set points encountered in household water heaters and allow for *L. pneumophila* growth.^39–42^

Extensive safety measures were implemented to prevent the release of Biosafety Level 2 (BSL-2) pathogens from the rig and allow inoculation with *L. pneumophila*. The system was moved to a BSL-2-certified laboratory and connected to an automated flood detection unit (Flood Master, Branford, CT). A backflow preventer and ball valve ensured that water supplied to the experimental apparatus did not reinfiltrate the building water system. Each pipe section was also equipped with an additional spring check valve (Sekisui, Taichung City, Taiwan) to prevent water flow from the pipes back into the water supply line and provide a barrier to cross contamination between pipes. The water exiting the flushing waste line was disinfected through two in-line ultraviolet (UV) light disinfection systems (VIQUA, Guelph, ON) in series before being heated at 65 °C for at least 10 min using a 22.7 L (6-gallon) water heater for further disinfection of host amoebae. This waste line was hard-plumbed into an existing building drain pipe.

The plumbing system was conditioned for 4 years to develop mature biofilms prior to the inoculation of pathogens. A strain of *L. pneumophila* isolated from Quincy, IL^43^ was inoculated 2 times from a separate rig system with water containing approximately 4000 MPN *L. pneumophila*/L, and inoculation was repeated in cold pipes after the room temperature was increased to 25 °C, as detailed in our previous study.^44,45^ All pipes were flushed once daily for 35 s, which is the median duration of use events for domestic hot water systems.^46^ Thus, the pipes were stagnant 99.96% of the time. During routine simulated water use events, the flow of water was controlled by automated timers (ChronTrol, San Diego, CA) and solenoid valves (ASCO Red Hat, St. Louis, MO).

Designed to evaluate a wide range of hydraulic conditions, the rig features flow rates from 0.95 to 8.3 L/min (0.25 to 2.2 gpm) and coils of pipe with diameters of 6.35, 12.7, and 19.05 mm (0.25, 0.5, and 0.75 in.). The WRT at the tap varied from 1 to 21.3 days, depending on the diameter and flow rate of the pipe. Each pipe had a sampling tap at the full length of the pipe as well as a tap at an intermediate length along the pipe, representing the distance water would travel after 1 day. In the case of pipes with an 8.3 L/min flow rate, the 1 day WRT tap was located at the full length of the pipe. For additional details see our prior publication describing the rig operation during its conditioning phase prior to *L. pneumophila* inoculation.^38,47^

All water entering the rig passed through four GAC filters in series during Stages I (18 °C room temperature) and II (25 °C room temperature), targeting a disinfectant residual of <0.2 mg/L. For subsequent stages, the portion of GAC-filtered water was strategically decreased to achieve influent chloramine levels of 0.25, 0.5, 1.0 mg/L and 2.5 mg/L while the room temperature remained at 25 °C. The rig was allowed to operate at each residual concentration for at least one month before sampling was conducted.

### Sample Collection and Analysis

#### Water Sampling Procedure

Procedures used to collect samples for each disinfection stage are detailed in our previous work.^47^ In brief, chemicals were analyzed during one sampling event, whereas biological sampling was conducted in three sequential sampling events 3 days apart using sterilized bottles and sodium thiosulfate (24 mg per liter of sample) for dechlorination. The three biological sampling events (considered triplicates) were staggered by 3 day intervals to allow water in all of the pipes to acclimate back to the design WRTs. The lag time needed between sampling events was calculated based on the volume of water drawn during sample collection and the volume of water flushed from each pipe daily.

#### Biological and Chemical Analysis

During each biological sampling event, culturable *L. pneumophila* were quantified using Legiolert potable most probable number (MPN) method (IDEXX, Westbrook, ME) undiluted or at 1:10 or 1:100 dilutions with autoclaved influent water serving as the diluent. Droplet digital polymerase chain reaction (ddPCR) was used at the end of the study to verify the presence of the *L. pneumophila* mip gene in positive wells of Legiolert samples collected from the rig cold and hot plumbing (Table S1 and S2). TCC were measured by staining samples with SYBR Green fluorescent nucleic acid before quantification using flow cytometry (BD Accuri C6; BD Biosciences, San Jose, CA) (SI 1). TCC (live and dead cells) was chosen as an analyte to evaluate cells as both growing microorganisms and a potential food source. An analysis of intact cell counts (ICC) was done with TCC at the 2.5 mg/L chloramine level to track necrotrophic growth in the rig (Figure S2; SI 2). Temperature was measured with a pH 150 m (Oakton Research, Vernon Hills, IL). Chloramine residual was measured as total chlorine using a DR3900 or DR5000 spectrophotometer with DPD (Method 8167, HACH, Loveland, CO). Additional aspects of the chemical water quality entering the pipes are detailed elsewhere.^47^

#### Glass Jar Growth Experiment

A glass jar experiment was conducted to evaluate the effects of temperature and disinfectant concentration on bacterial growth in the test water in the absence of plumbing. Drinking water samples were collected from the rig influent 11 months after the start of the rig experiment and incubated in 18 nitric acid-washed and nanopore water rinsed 250 mL amber glass jars. Jars were subsequently baked in a muffle furnace at 550 °C for 5 h. Half of the jars received chloraminated water directly from the tap, which happened to have 5.70 mg/L Cl_2_ on the day of testing. The remaining jars were filled with GAC-filtered water with a 0.04 mg/L total chlorine residual. Samples were stored in triplicate at 25 °C, 30 °C, and 37 °C concealed from light, and TCC in the jars were monitored over 35 days using flow cytometry.

#### Statistical Analysis

R Studio version 4.2.3. was used to perform statistical tests. Microbial abundance in the water heater was fitted using a linear model. A nonparametric Spearman rank correlation was used to compare TCC between the rig influent and water heater effluent. An alpha of 0.05 was set as the threshold for significance except for paired Wilcoxon Rank Sum tests for temperature in glass jars where alpha was set as 0.017 based on the Bonferroni correction which compensates for multiple comparisons between the three temperature conditions.

## Results and Discussion

### Microbial Growth Curve in Glass Jars

Unchloraminated jars, i.e., with chloramine removed from the town water supply by passing through a GAC-filter, initially contained a TCC of 5 log cells/mL and increased by a half log cells/mL within 3 days of incubation, without any observed lag (Figure 2). TCC in jars stored at 25 and 30 °C behaved similarly, eventually peaking around 5.7 log cells/mL after 9–14 days before decaying about 0.25 log cells/mL (Figure 2). Though the TCC curve was not statistically different from 25 °C (paired Wilcoxon Rank Sum, *p*-value = 0.910) or 30 °C (paired Wilcoxon Rank Sum, p-value = 0.496), TCC reached the stationary phase at 3 days only in the 37 °C jars, indicating that temperature influenced nutrient assimilation, bacterial competition, and optimal growth conditions. For instance, it is reported that the optimal growth temperature for nitrifying bacteria is 20–30 °C.^48–51^ This is consistent with other studies that described temperature-dependent colonization and growth of drinking water taxa, including OPs, with differing growth rates.^52–54^

**Figure 2:**
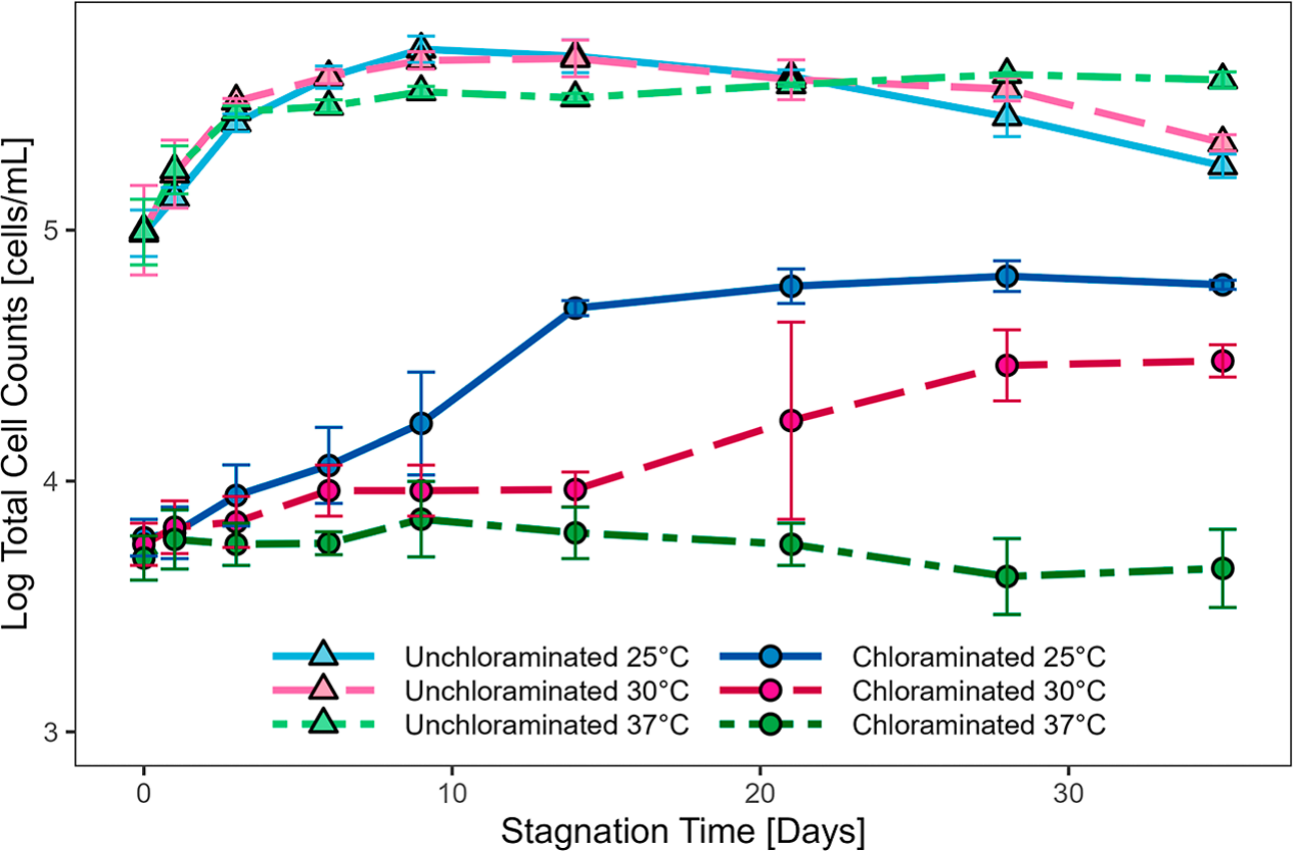
Total cell counts (TCC) of chloraminated (5.70 mg/L as total Cl_2_) and unchloraminated via GAC filtration (0.04 mg/L as total Cl_2_) tap water in glass jars as a function of stagnation time. Error bars represent standard deviation of replicate jars (*n* = 3).

The chloraminated jars, which receive water that had not passed through the biologically active GAC filters, initially contained ∼1.25 log cells/mL less TCC than the unchloraminated jars (Unpaired Wilcoxon Rank Sum, *p*-value = <0.001) (Figure 2). After 30 days of incubation, it was surprising to find that a total chlorine residual of 0.33 ± 0.05 mg/L still remained in all three of the 37 °C jars, which had essentially no cell growth, while the chloramine was nondetectable in the 25 and 30 °C jars. This aligns with the finding that chloramine in the Blacksburg, VA tap water decayed slower at ∼37 °C compared to lower ambient temperatures (19–30 °C), presumably due to reduced nitrifier activity at the higher temperatures.^47^ The 25 and 30 °C jars experienced phases of rapid growth to a plateau. Notably, a decline in growth still was not evident by the time the experiment ended at 35 days. Overall, these observations are consistent with the hypothesis of growth being suppressed or suspended when chloramine is present, with the phases of microbial growth commencing once it is depleted.

### Chloramine and Temperature in the Rig

At the cold 1 day WRT taps, hourly monitoring of small aliquots taken from within the stagnant pipes after flushing 2–3× pipe volumes revealed that about 92% of the chloramine residual was lost within 4 h, irrespective of influent chloramine concentration.^47^ Chloramine decayed 2–3 orders of magnitude faster in the rig pipes in contrast to the same water held in glass jars,^47^ illustrating how the realities of mature biofilm, pipe material, and sediment in real-world plumbing can undermine disinfection efficiency.^55–60^ Because of this, chloramine levels at all hot and >1 day cold WRT pipe taps were consistently below the WHO recommended minimum building influent chlorine residual of 0.2 mg/L.^61^

Cold water entering the rig varied seasonally, with a temperature range of 14–24 °C over the course of the 2 year study. At a water heater set point of 40 °C, the water feeding the hot pipes was consistently between 33 and 35 °C. No *L. pneumophila* growth was detected in the cold water pipes at the lower room temperature of 18 °C, which is considered to be within the dormant or slow growth temperature range for *Legionella* and aligns with previously reported *L. pneumophila* growth thresholds.^42,62,63^ However, we observed *L. pneumophila* growth in the cold water pipes when the ambient room temperature was warmed to 25 °C.

### Bacterial Growth Profiles in the Rig

#### Cold Water Plumbing

Some seasonal variability in rig influent TCC concentrations was observed over the duration of the study, which is typical for drinking water derived from a surface water source (Figure S3). In addition to controlling the influent chloramine level, GAC filtration was used to simulate the conversion of biodegradable organic carbon to biomass that can occur within a DWDS.^18,64^ Seasonal variability and GAC filtration also caused slight changes in total organic carbon (Table S3). Despite this variability in influent water, TCC at the cold taps remained consistent across stages (Figure 3a). For all five influent chloramine levels, the greatest log growth occurred within the first day. Furthermore, the peak TCC tended to occur in the 8.82 day WRT tap for all stages. The peaks were consistently 5.67–5.90 log cells/mL, regardless of influent TCC or chloramine level. Interestingly, this is about the same peak concentration and time observed in the complementary glass jar experiment at 25 °C without chloramine (5.73 log cells/mL after 9 days) (Figure 2). The similitude between the glass jars without chloramine and pipes was surprising, because the pipes contained biofilms that had matured over a 4–6 year period, whereas the glass jars would have only contained minimal biofilm that was able to colonize the inert material over the course of the one-month experiment. By contrast, the 25 °C glass jars with persistent chloramine contained 0.97 log cells/mL less TCC than in the pipes with high (though not persistent) influent chloramine. A portion of this difference is because the 25 °C glass jars started at an average TCC 0.18 log cells/mL less than the lowest TCC measured at the rig influent. Importantly, there was also 19–32% decay in TCC in the rig at the oldest WRT of 17.39 days.

**Figure 3:**
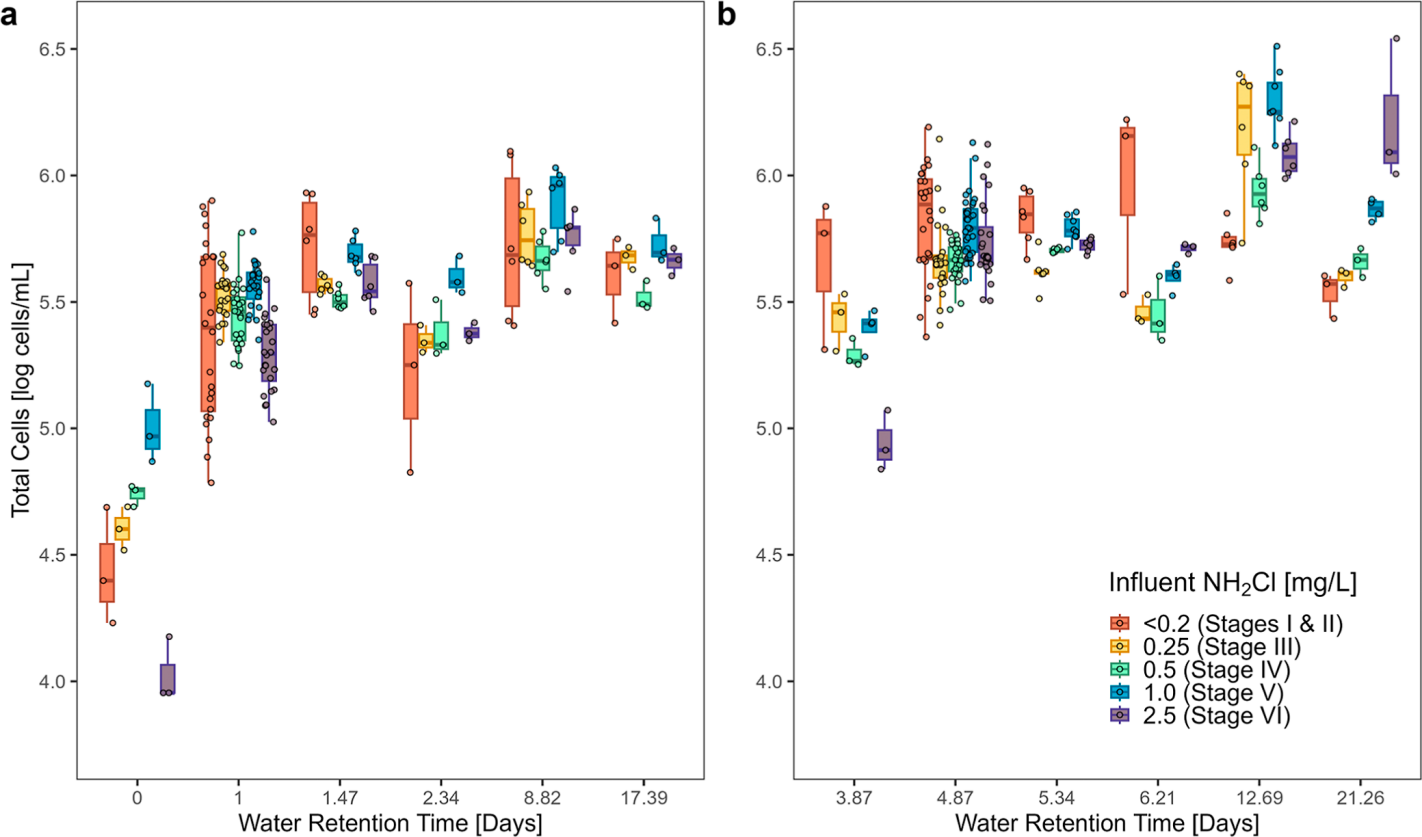
Total cell counts (TCC) measured at (a) cold water and (b) hot water sampling ports in the plumbing rig. Water retention time of 0 days: blend of unfiltered and GAC-filtered cold water entering the cold pipes and water heater tank. Water retention time of 3.87 days: the hydraulic retention time of the water heater tank (40 °C set point) which supplied the hot pipes was included in the WRT calculation of each hot water tap. Boxplots represent the 25th, 50th, and 75th percentile; whiskers represent 1.5 times the interquartile range. Total sample numbers varied by WRT and individual data points overlay the boxplots. The rig and corresponding biofilms were aged 4–6 years over the course of this experiment.

#### Hot Water Plumbing

Understanding flow within water heaters is vital to characterize the level of thermal or chemical inactivation they provide in terms of disinfection. Hydrodynamics within a conventional tank water heater are expected to mainly be a function of shape, size, temperature settings and flow pattern into the tank.^65–69^ The level of mixing that occurs in the tank influences the retention (i.e., disinfectant contact) time available. Large water heaters with multiple heating elements have sometimes shown stratification based on temperature set points and tank efficiency.^56,65,66,68,69^ However, in another study employing a fluoride tracer on 189 L (50-gallon) water heaters at room temperature and 49 °C, it was found that a continuous stirred tank reactor (CSTR) model accurately represented flow behavior.^67^ If that is the case, we would expect that a water heater with a set point of 60 °C would kill most drinking water associated organisms, effectively resetting the biological water age clock and microbial growth curve back to zero days.^70–74^

We previously cited chloramine data to argue that the tank used in this study was also behaving as a CSTR within the first hour of the flush cycle.^44,45^ The average WRT in the heater effluent was 3.87 days based on the volume of the tank and flow. For this study, since the water heater set point was below the temperature required to kill most drinking water bacteria (40 °C set point), the 3.87 day retention time of the tank was added to the retention time of water in the hot pipes for the data analysis and plots presented herein.

As the chloramine level in the rig influent increased, the median TCC in the water heater effluent decreased by 0.86 log cells/mL. Importantly, the difference in TCC between the rig influent and water heater effluent varied slightly by stage due to seasonal variability and GAC filtration of the influent (Figure S4; Spearman rank sum, n = 15, rho = 0.43, p-value = 0.112). During Stages I and II, when influent chloramine concentrations were lowest, most of the bacterial growth occurred in the water heater relative to TCC in the rig influent (Figure 3b). Microbial decay was apparent in the pipes as TCC decreased by a half log cells/mL from the peak at 6.06 log cells/mL. In stark contrast, as the chloramine concentration in the influent increased during Stages III through VI, there was a period of exponential growth after the water heater and less decay at older WRTs. Likewise, the WRT of peak growth shifted from 6.21 to 21.26 days as influent chloramine increased, though the magnitude of peak growth remained within a range of 0.36 log cells/mL for all stages. Hence, the chloramine residual delayed or partly suspended the growth in the water heater, allowing the growth curve to resume after chloramine was depleted in the pipes.

Interestingly, TCC in the water heater effluent during Stage VI, with 2.5 mg/L as Cl_2_, was in similar range as TCC in the cold rig influent, demonstrating that disinfectant in the tank held TCC at a level consistent with cold water at day zero. Reanalysis of data from an earlier study conducted on the rig revealed that increasing the water heater set point from 40 to 60 °C led to a 0.61 log cells/mL decline in TCC in the water heater (Figure 4), consistent with our proposed concept of a biological water age “re-set” event. The addition of 1.2 mg/L influent chloramine at 60 °C resulted in a further 0.20 log cells/mL decrease in TCC in the tank, despite a 0.07 log cells/mL increase in TCC in the influent (data not shown). However, TCC still followed the expected microbial growth trend in pipes after chloramine was no longer detectable in the pipes.^38^ These changes in growth profiles and peak WRT support the hypothesis that as disinfectant dose increases, the microbial growth curve will be delayed until chloramine decays.

**Figure 4:**
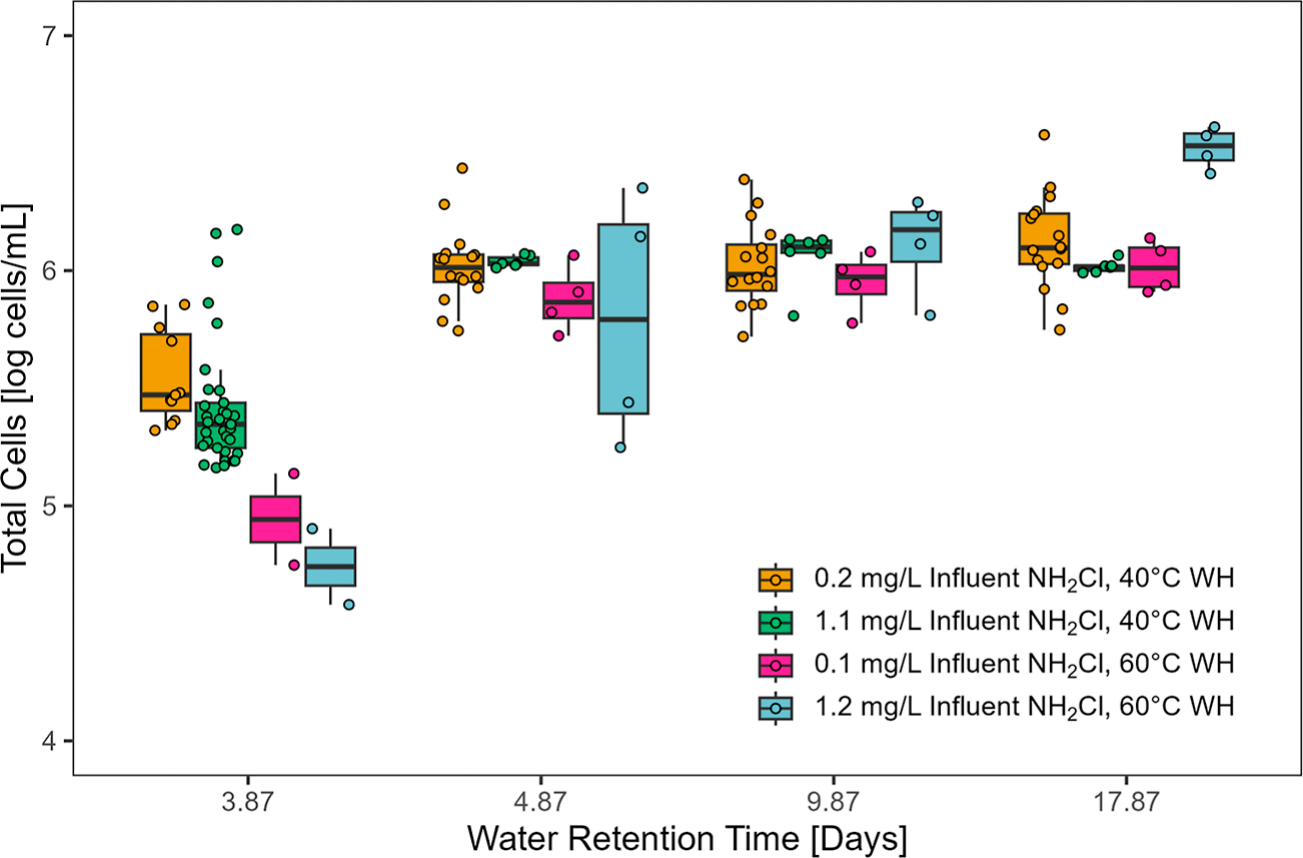
Total cell counts (TCC) in hot water plumbing at a 25 °C room temperature and rig age of 1.5 years. Water retention time of 3.87 days: the hydraulic retention time of the water heater tank which supplied the hot pipes was included in the retention time of each hot water tap. Boxplots represent the 25th, 50th, and 75th percentile; whiskers represent 1.5 times the interquartile range. Total sample numbers varied by water retention time, and individual data points overlay the boxplots. WH: water heater set point. Raw data was derived from previous study^38^ and reanalyzed to generate this figure.

#### *L. pneumophila* Growth Profiles in the Rig

Directly enumerating *L. pneumophila*, or the broader genus *Legionella*, is costly, especially when employed for the routine monitoring of building water systems.^75^ This has spurred interest in examining heterotrophic plate count (HPC) bacteria as an indicator of *Legionella* occurrence.^76–78^ However, HPCs only capture a small fraction of the microbiome and there are distinct differences in microbial ecology between *Legionella*, HPCs, and other drinking water-associated bacteria.^24^ Overall, HPCs have been found not to be predictive of *Legionella* and thus the National Academies of Science, Engineering, and Medicine (NASEM) recently advised against monitoring HPCs for this purpose.^79^ Above, we established the behavior of TCC in the pipe rig, which more broadly captures cellular abundance than HPCs, because it is a direct cell count and does not rely on microbial culture. Below we examine the extent to which *L. pneumophila* growth mirrored that of TCCs and whether it also followed the classic microbial growth curve in the plumbing rig.

##### Cold Water Plumbing

*L. pneumophila* was never detected in the influent water to the rig. Therefore, any changes in *L. pneumophila* concentrations in the cold water pipes were a result of growth or decay following inoculation. At 0.5 mg/L influent chloramine, there was no detectable *L. pneumophila* at most of the 1 day WRT taps. When the residual was elevated to 1 mg/L or above, *L. pneumophila* was never detected in any of the 1 day WRT taps (Figure 5a). However, *L. pneumophila* was persistent in all taps at WRT beyond 1 day, even up to the highest influent chloramine concentration of 2.5 mg/L. This survival is likely related to the finding that a majority of chloramine decayed within only 4 h in pipes at 1 day WRT. *L. pneumophila* concentrations plateaued around 1 MPN/mL, with little or no (0–52%) decay in *L. pneumophila* at higher WRT. We speculate that the pipe biofilms reached an effective carrying capacity due to limitations in available nutrients for *L. pneumophila* growth. While a majority of *L. pneumophila* growth in cold pipes occurred within 24 h, similar to TCC, *L. pneumophila* was found to be poorly correlated to TCC (R^2^ = 0.27, p-value = <0.001).

**Figure 5:**
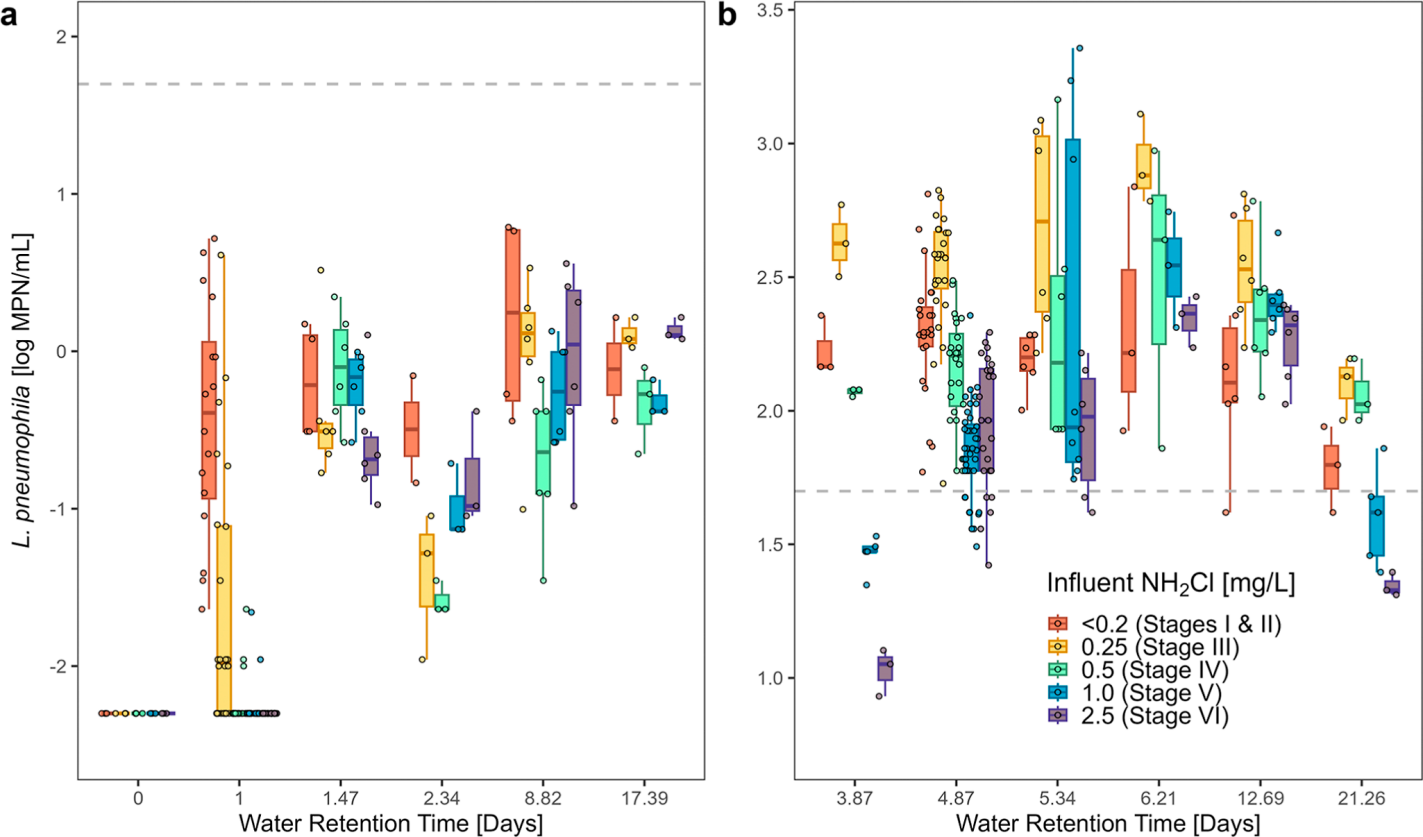
*Legionella pneumophila* abundance in (a) cold water and (b) hot water plumbing samples at a rig age of 4–6 years. Water retention time (WRT) of 0 days: blend of unfiltered and granular activated carbon (GAC) filtered cold water entering the cold pipes and water heater tank to achieve target influent disinfectant concentration. WRT of 3.87 days: the WRT of the water heater tank (40 °C set point) that supplied the hot pipes was included in the WRT calculation of each hot water tap. Boxplots represent the 25th, 50th, and 75th percentile; whiskers represent 1.5 times the interquartile range. Total sample numbers varied by the tap corresponding to each WRT, and individual data points overlay the box plots. Horizontal dashed lines represent the NASEM *Legionella* action level of 50 CFU/mL.^79^ Note the difference in *y*-axis scale between panel a and b, to aid in visualizing the microbial growth curve for the cold and hot water, respectively.

#### Hot Water Plumbing

At all levels of influent disinfectant, the water heater served as a continual source of *L. pneumophila* seeding the hot water pipes (Figure 5b). When influent disinfectant concentrations were low (≤0.25 mg/L) (Stages I, II, and III), 51–79% of the peak *L. pneumophila* abundance was reached in the water heater itself. Levels of *L. pneumophila* in the water heater significantly decreased as the concentration of chloramine in the tank increased throughout the experiment, consistent with chloramine inactivation of *L. pneumophila* in the tank (n = 17, p-value = <0.001). The *L. pneumophila* concentration in the tank effluent decreased at approximately 0.606 log MPN/mL per mg/L of chloramine in the rig influent. It is hypothesized that water leaving the tank when the influent disinfectant dose was high (≥1.0 mg/L) (Stages V and VI) still contained readily available nutrients, but very little chloramine, shifting the majority (91–95%) of *L. pneumophila* growth to the pipes rather than the water heater. During all disinfection stages, *L. pneumophila* peaked at the 6.21 day WRT tap, and decay dominated thereafter, with a 63–90% drop in *L. pneumophila*.

The peak *L. pneumophila* in the hot pipes was 2.5 log MPN/mL higher than that observed in the cold pipes. In cold water, *L. pneumophila* never exceeded 1 log MPN/mL, whereas numbers were nearly always >1 log MPN/mL in hot water locations. However, as influent chloramine was elevated, *L. pneumophila* abundance in the highest WRT hot water pipe approached within 15 MPN/mL of the concentrations observed in the cold water pipes. Concerningly, *L. pneumophila* levels in the hot water plumbing only consistently fell below the NASEM *Legionella* action level (50 colony forming units (CFU)/mL, assuming that MPN approximates CFU) in the water heater and at the highest WRT tap once influent chloramine was at least 1 mg/L (Figure 5b).^79^ Therefore, in the scenario tested by the at scale plumbing rig, where influent disinfectant residuals are low and further depleted by the water heater, weekly flushing can actually stimulate *L. pneumophila* growth. Effectively, rather than washing out the *L. pneumophila*, flushing acts to introduce nutrients and enable *L. pneumophila* to maintain near peak growth as opposed to entering a decay phase. Consistent with this hypothesis, Rhoads et al.^80^ attributed *a* > 1.5 log MPN/mL decrease in *L. pneumophila* after 3–7 weeks of stagnation within a building to nutrient limitation. They further observed that follow-up flushing increased *L. pneumophila* by 1–2.5 log MPN/mL.

### Hydraulic Design Characteristics Did Not Directly Impact Microbial Growth

Adjusted WRT was found to be a master variable controlling microbial growth trends, rather than the extremes in flow velocity and pipe diameter examined in this study. Beyond controlling WRT, differences in flow rate, flow velocity, and pipe diameter played relatively little role in TCC or *L. pneumophila* growth in the rig under the operational conditions tested during this experiment (Figure S5 and S6). Though hydraulics have been reported to have a limited impact on bulk water cell counts in a pilot- and full-scale DWDS,^18,81^ we consider it possible that hydraulics could play a much larger role in building plumbing if testing was conducted with greater flush durations and frequencies, an experiment which would be of interest in future studies. It is further important to point out that the relationship between WRT and bulk water TCC was not simply linear or exponential. From this standpoint, the microbial growth curve provides a useful mechanistic framework for predicting *L. pneumophila* behavior in building plumbing as a function of biological water age.

### A Compelling Case for the Microbial Growth Curve Framework

This study systematically demonstrated shortfalls in the conventional wisdom of microbial and OP growth directly correlating with hydraulic water age or WRT. We propose the classic microbial growth curve as an alternative framework that can be shifted and modified based on influent disinfection conditions, water heating, and other relevant water system design, control, and operational factors (Figure 1). We found that trends in TCC and *L. pneumophila* growth profiles were remarkably reflective of the microbial growth curve framework, exhibiting consistency across both hot and cold water rigs, a range of hydraulic conditions, and low to high influent disinfectant. Contrary to expectations, factors such as hydraulics had little to no direct effect on observed growth trends. Instead, the concentration of disinfectant residual and proximity to upstream water heating influenced whether lag, exponential, stationary, or death/decay dominated in downstream pipes.

When nutrients are in excess, once acclimated, cells will typically enter an exponential growth phase. As nutrients become depleted, the stationary phase ensues, in which growth and death rates are approximately equivalent. During this phase, energy production and cell division decrease for *L. pneumophila*, as they do for bacteria in general.^82–85^ Hence, TCC abundance in cold water plumbing usually displayed exponential growth only during the first day for all influent chloramine conditions and maintained the stationary phase thereafter (Figure 6a). *L. pneumophila* behavior in the cold plumbing did not exactly mirror that of TCC, likely because *L. pneumophila* was never detectable in the water entering the rig and therefore the exponential growth phase could not ensue, even at the trace influent chloramine concentration (<0.2 mg/L) (Figure 6e).

**Figure 6:**
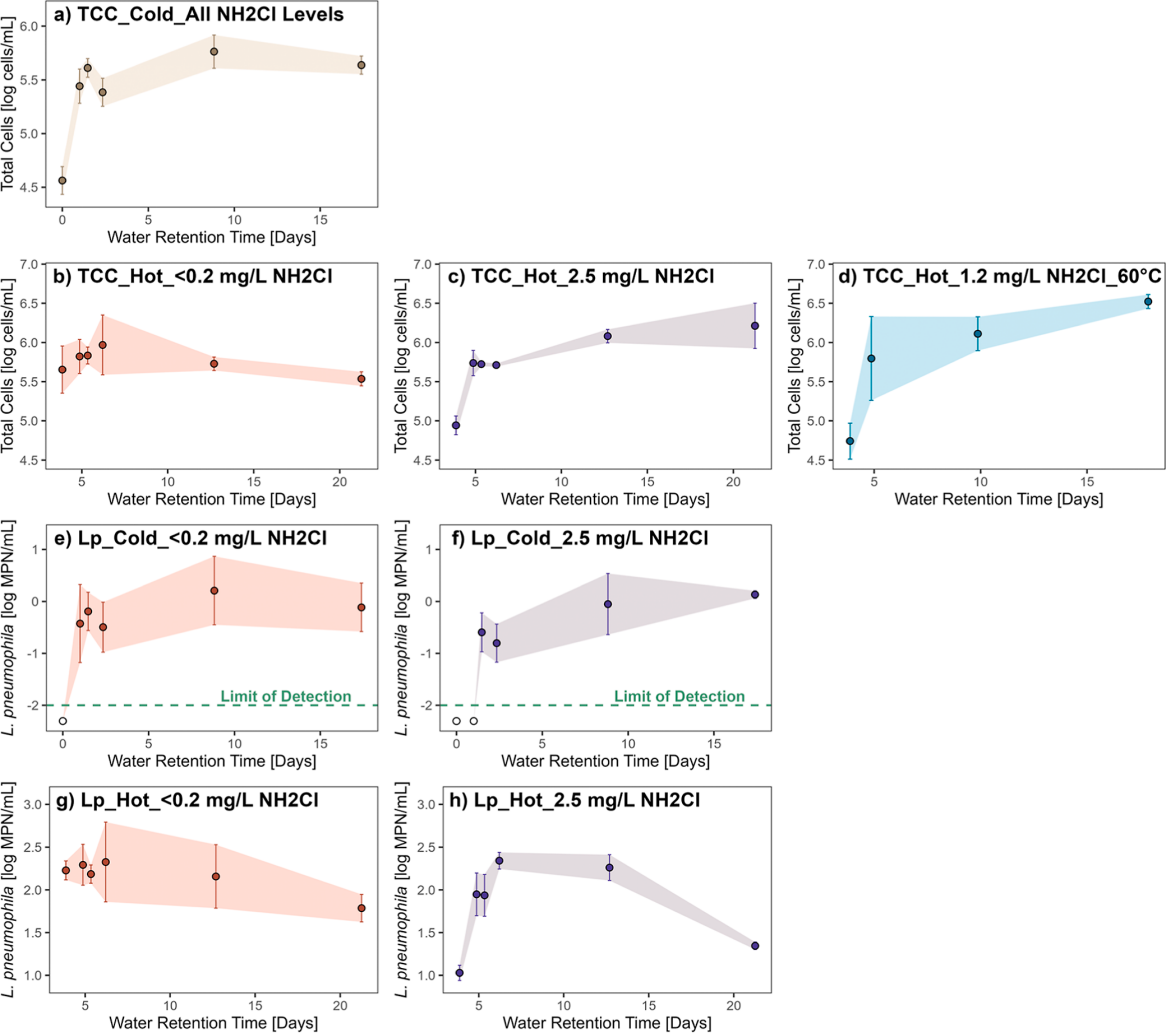
Extremes in chloramine and heat disinfection tested in the pipe rig. Total cell counts (TCC) profiles in (a) cold water, hot water at (b) <0.2 mg/L NH_2_Cl and (c) 2.5 mg/L NH_2_Cl influent, and (d) hot water with 1.2 mg/L influent NH_2_Cl and a water heater set point of 60 °C. *Legionella pneumophila* abundance profiles in cold water with (e) <0.2 mg/L NH_2_Cl and (f) 2.5 mg/L NH_2_Cl influent and hot water with (g) <0.2 mg/L NH_2_Cl and (h) 2.5 mg/L NH_2_Cl influent. Total sample numbers varied by water retention time (WRT) with *n* = 3–39. Error bars represent standard deviation for replicate samples. Raw data for the 60 °C set point was collected by Busch et al.^38^ using the same rig at 1.5 years age and reanalyzed together with the data collected in this study.

In the hot water pipes, given that the warm temperature of 40 °C is ideal for growth of a wide range of microbes,^62^ water leaving the tank during the trace influent chloramine condition had already exerted the majority of their growth potential. Subsequently, levels of both TCC and *L. pneumophila* in the hot water pipes were stable or eventually decayed during the trace disinfectant stage (Figure 6b and g). Accordingly, we previously reported a 3 log MPN/mL decrease in *L. pneumophila* abundance in polyvinyl chloride + iron pipe segments that were stagnant for one year, providing evidence that microbial growth can eventually peak and decay (data not shown). There is potential for *L. pneumophila* to persist by entering a viable but nonculturable state during starvation, which warrants further study as a contribution to decay in culturable *L. pneumophila* signal.^85,86^

In several cases, disinfectant acted to slow or reset the biological water age clock in the rig by suppressing growth. After *L. pneumophila* was diminished in the cold 1 day WRT taps, the start of the stationary phase shifted to a WRT where chloramine was absent (Figure 6f). In the hot water plumbing, higher influent disinfectant levels and elevated temperature inhibited TCC and *L. pneumophila* growth in the water heater, shifting the lion’s share of exponential growth phase to the downstream pipes (Figure 6c,d, and h). Prior studies have demonstrated how dead biomass, resulting from chemical or heat disinfection, can provide nutrients for regrowth of *Legionella* via necrotrophy.^87,88^ As such, nutrients from killed cells likely contributed to downstream growth in the rig. The lag phase was not able to be isolated at the time scale measured here, as chloramine residuals decayed and heat was lost within 4 h after entering pipes.^38,47^ Consistent with other studies,^91–94^ no level of applied treatment was effective at completely eliminating *L. pneumophila* or bacterial growth in the rig. As discussed elsewhere,^80^ these results highlight the fallacy that preventing *stagnation* is equivalent to reducing *water age*. Furthermore, it is important to recognize that increasing use frequency does not necessarily result in complete displacement of water in a system.^8,11,95–98^ For example, the stagnation time in the pipe rig was fixed at 24 h, yet hydraulic water age was still >17 days at some taps. Overall, this study provides mechanistic insight into why flushing can fail to mitigate *Legionella* growth in buildings and can actually amplify the risk, especially in waters that do not carry a disinfectant residual.

Based on the findings of this study, we propose a microbial growth curve framework for understanding and predicting growth potential of *L. pneumophila* and other OPs in building plumbing. This framework extends the theoretical principle put forth by Favere et al.^99^ of promoting biostability by striving to maintain an active biomass to available nutrient ratio at the carrying capacity, thus avoiding potential for exponential growth. Here, the microbial growth curve framework expands this principle across the full spectrum of microbial growth phases, empirically demonstrating it in premise plumbing scenarios using data collected from an at-scale rig, and going beyond biostability to evaluate response of *L. pneumophila*, a key OP of concern with distinct ecology relative to other heterotrophic drinking water microbiota. The microbial growth framework can be applied toward considering engineering design, control, and operational conditions in light of where along the treated drinking water to tap continuum exponential and stationary growth are expected to occur and estimating the maximum growth potential. In other words, the microbial growth curve and its magnitude can be intentionally shifted to optimally mitigate OP growth and risk of consumer exposure at the tap. Depending on nutrient availability and disinfectant presence and persistence, we expect that every water system can be characterized by a “worst-case” microbiota-specific biological water age at which a peak microbial number are reached and before cell decay begins to dominate as nutrient limitations set in. In this study the worst-case biological water age tended to occur at about a 1 week WRT. The findings lend caution to assuming that routine and costly flushing regimes universally reduce microbial risk. Rather, development of building-specific water management plans should incorporate influent water characteristics, including nutrient availability (e.g., oxygen,^23^ biodegradable organic matter,^100^ and phosphorus^101^) and disinfectant stability, to determine whether a given flushing frequency will produce beneficial or adverse outcomes. Findings from this research indicate the existence of a worst-case flushing frequency that intermittently introduces nutrients to premise plumbing while failing to sustain a disinfectant residual adequate for controlling microbial proliferation. Previous assumptions that increasing flushing universally improves water quality failed to consider the potential influence of endogenous decay or nutrient-limited growth conditions. Similarly, these results suggest that extended stagnation in unoccupied buildings may not inherently elevate health risks, highlighting opportunities to conserve labor and water resources without compromising public health protection.

## Conclusions

This study reveals that the trends of a classic microbial growth curve, including a cell decay phase, can apply in building plumbing and serve as a framework to understand pathogen and bacterial growth. Here we provide an in-depth evaluation of bulk water TCC and *L. pneumophila* growth profiles within building plumbing in response to chloramine and temperature, using a pipe rig to systematically examine these factors, in parallel, under a range of relevant real-world hydraulic conditions. Among the evaluated variables, WRT only stood out as a dominant factor controlling growth dynamics in the rig when adjusted for disinfectant levels and upstream water heating and also recognizing that microbes eventually enter a death/decay phase once nutrients are depleted. Increasing the influent chloramine helped to mitigate *L. pneumophila* growth in some cases but effectively pushed exponential growth potential downstream as disinfectant decayed. Notably, *L. pneumophila* was found to persist in both the hot and >1 day WRT cold taps, despite an influent chloramine residual up to 2.5 mg/L. Thus, influent disinfectant concentration is only one dimension of effectively controlling OPs in building plumbing.

## Supplementary Material



## Notes

At-scale premise plumbing rig schematic; *Legionella* spp. and *L. pneumophila* ddPCR multiplex assay information and references; Stage VI *Legionella* spp. and *L. pneumophila* ddPCR results; Flow Cytometry Staining Procedure; Total and Intact Cell Counts at Cold and Hot Taps; Total and Intact Cell Count Discussion; Seasonal variability in influent cell counts; Impact of seasonal variability and GAC filtration on TOC; Comparison of total cells in water at the rig influent and water heater effluent; Impact of Hydraulic Design Characteristics in Cold Water Pipes; Impact of Hydraulic Design Characteristics in Hot Water Pipes (PDF)
