# Supplementary material for: Microbial Growth Curve Framework Provides Insights for Controlling Opportunistic Pathogens in Building Plumbing
Source: ACS ES T Water. 2026 Apr 10;6(5):2937–48. doi: 10.1021/acsestwater.5c01431 (PMC13162260; doi:10.1021/acsestwater.5c01431)
Supplement: Supplementary file 1 [file ew5c01431_si_001.pdf]

Supplemental Information for

MICROBIAL GROWTH CURVE FRAMEWORK PROVIDES INSIGHTS FOR CONTROLLING OPPORTUNISTIC PATHOGENS IN BUILDING PLUMBING

Tolulope O. Odimayomi[1]*, Amy Pruden[1], Marc A. Edwards[1]

[1]Via Department of Civil and Environmental Engineering, Virginia Tech, Blacksburg, Virginia 24061, United States

*Email: todimayo@vt.edu

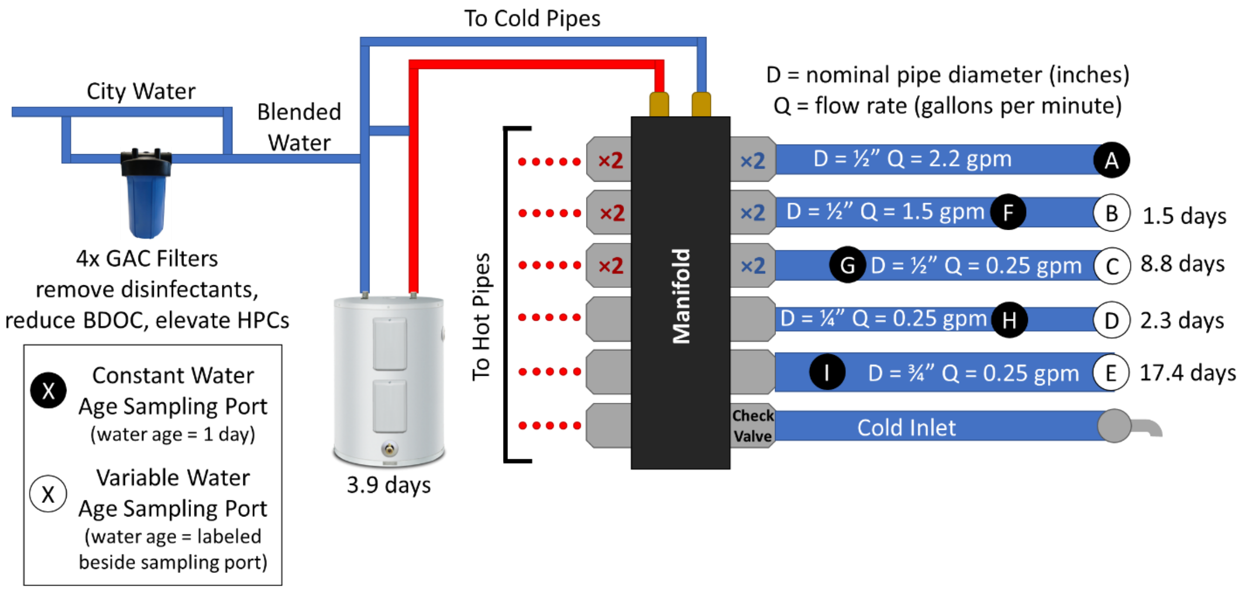

Figure S1. At-scale premise plumbing rig schematic. Identical cold and hot water pipe branches examine the effect of varying pipe diameter, flow rate, and water retention time (WRT) on disinfectant residuals and opportunistic pathogen growth. The top three pipe segments A, B and C are duplicated, resulting in a total of 16 cold and hot pipe branches. Sampling ports are installed at strategic distances along each pipe to reflect important water use and design scenarios of constant 1-day WRT (black circles) or at the end of the pipe with variable WRT (white circles). Reprinted with permission under a Creative Commons CC-BY 4.0 license from Odimayomi et al.[1] Copyright © 2026 The Authors. Published by American Chemical Society.

Table S1. ***Legionella*** **spp. and** ***Legionella pneumophila*** **ddPCR multiplex assay information and references**

Targeted organisms	Targeted genes	Sequences (5'-3')	Amplicon (bp)	Enzyme activation/ deactivating	Denaturation / annealing / extension	Reference
Legionella spp.	23S rRNA	Leg23SF: CCCATGAAGCCCGTTG AA<br>Leg23SR: ACAATCAGCCAATTAG TACGAGTTAGC<br>Probe: HEX-TCCACACCTCGCCTAT CAACGTCGTAGT/3BHQ -1	92	95 °C for 10 min / 98 °C for 10 min	40 cycles of 94 °C for 30 s and 57.1 °C for 60 s	(Nazarian et al., 2008)[2]
L. pneumophila	mip	Forward Primer: AAAGGCATGCAAGAC GCTATG<br>Reverse Primer: GAAACTTGTTAAGAAC GTCTTTCATTTG<br>Probe: FAM-TGGCGCTCAATTGGCT TTAACCGA/BHQ1	78			

Each 20 µL reaction contained 5 µl of molecular grade water, 10 µl of Probes ddPCR Supermix (Bio-Rad), 900nM each primer for both *Legionella* spp. and *L. pneumophila*, 250nM probe for both *Legionella* spp. and *L. pneumophila*, and 3 µl of DNA template.

Table S2. Stage VI *Legionella* spp. and *Legionella pneumophila* ddPCR results

Pipe Diameter (in), Flow Rate (gpm), Replicate‡	Plumbing Type	Water Retention Time (days)	Log *L. pneumophila* (gc/mL)*	Log *Legionella* spp. (gc/mL)*
1/2", 1.5 gpm, R1	Cold Water	1.47	8.06	8.51
Water Heater	Hot Water	3.87	7.78	8.20
3/4", 0.25 gpm	Hot Water	21.26	7.89	8.29

‡The ability to replicate pipe conditions was limited by plumbing manifold ports.

*DNA was extracted from presumptive positive (brown) large wells from Legiolert trays that had been incubated at 39 °C for 7 days as a test for *L. pneumophila* presence-absence.

SI 1. Flow cytometry staining procedure

SYBR Green I (10,000× concentrate in dimethyl sulfoxide (DMSO), Invitrogen, Carlsbad, CA) was diluted 100 times in DSMO (ReagentPlus, Sigma-Aldrich, St. Louis, MO). Samples were stained with 10 µl staining solution/mL sample and incubated for 10 min at 37 °C before quantification using flow cytometry (BD Accuri C6; BD Biosciences, San Jose, CA).

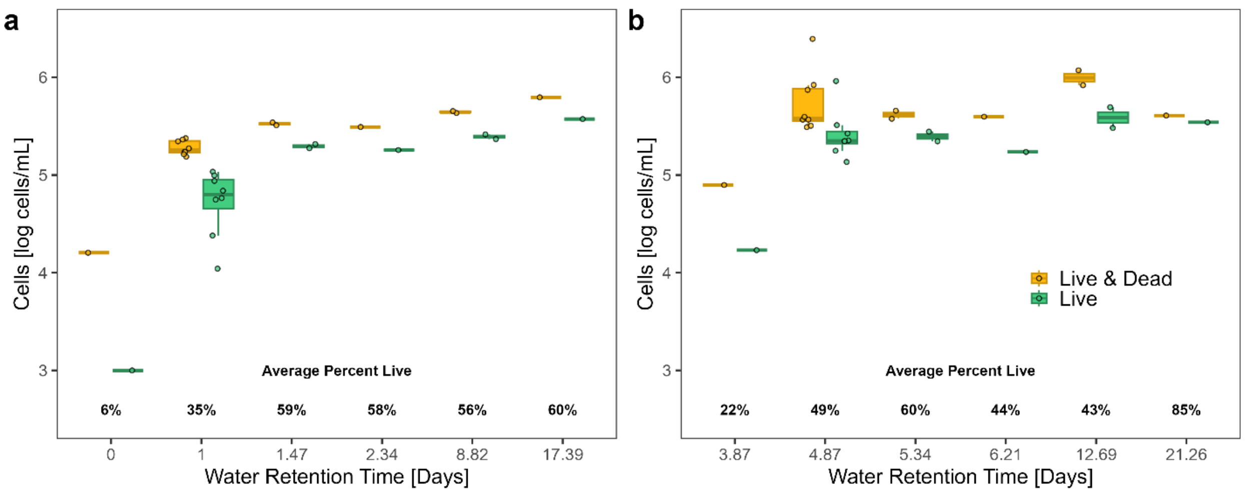

Figure S2. Total and intact cell counts at cold and hot taps. Total (TCC) and intact (ICC) cell counts measured at a) cold water and b) hot water sampling ports in the plumbing rig after 10 months at an influent chloramine level of 2.5 mg/L. Water retention time (WRT) of 0 days: blend of unfiltered and granular activated carbon (GAC)-filtered cold water entering the cold pipes and water heater tank. Water retention time of 3.87 days: the hydraulic retention time of the water heater tank (40 °C set point) which supplied the hot pipes was included in the WRT calculation of each hot water tap. Boxplots represent the 25th, 50th, and 75th percentile; whiskers represent 1.5 times the interquartile range. Individual data points overlay the boxplots.

SI 2. Total and intact cell count discussion

Total and intact cell counts (TCC and ICC) were measured in the plumbing rig 10 months after the rig had been receiving an influent chloramine dose of 2.5 mg/L (Figure S1a). In the cold water plumbing, there was slight increase in TCC and ICC after the 1-day WRT with the average percentage of live cell remaining at 56-60% compared to 6% in the rig influent water. For the hot water plumbing, on the other hand, TCC peaked at the 12.69-day WRT with 43% of those cells being intact (Figure S1b). While TCC decreased at the oldest WRT, ICC remained constant resulting in 85% of cells being intact at the 21.26-day WRT. This trend between TCC and ICC strengthens the conclusion that necrotrophic growth can occur once nutrients are limited.

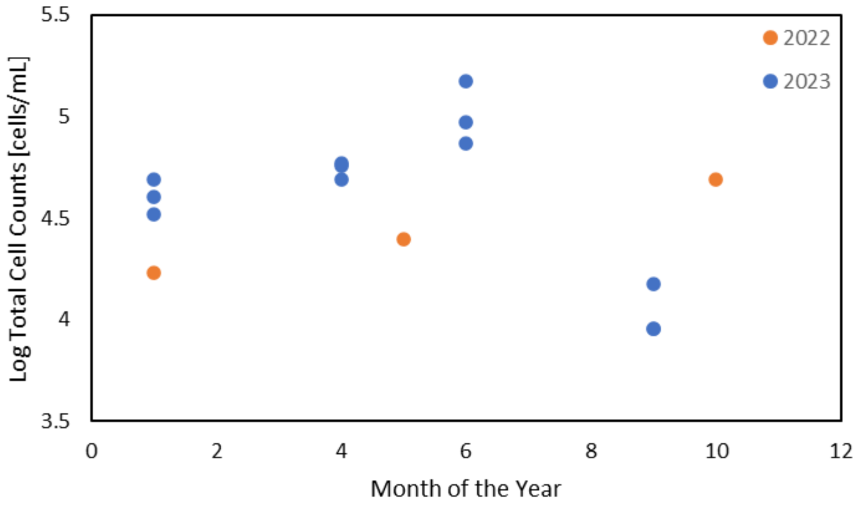

Figure S3. Seasonal variability in influent cell counts. Total cell counts (TCC) of rig influent water shifted seasonally and from year to year.

Table S3. Impact of seasonal variability and GAC filtration on TOC#

		TOC (mg/L)*	
**Month**	**Date**	**Municipal Tap Water**	**GAC Filtered Water**
May	2023-05-22	0.861	0.679
June	2023-06-07	0.839	0.596
July	2023-07-03	1.122	0.730
August	2023-08-02	1.029	0.737
September	2023-09-07	1.075	0.622
November	2023-11-16	1.051	0.820
December	2023-12-14	1.054	0.882

#Samples were collected from the Blacksburg, VA tap water entering the plumbing rig and a similar GAC filtration system to the one that supplied the rig.

*Total organic carbon (TOC) was measured using a Sievers 5310 Laboratory TOC-MS Analyzer (Suez Water Technologies, Trevose, PA, USA) or TOC-L_{CSN} Standard Model (Shimadzu, Japan).

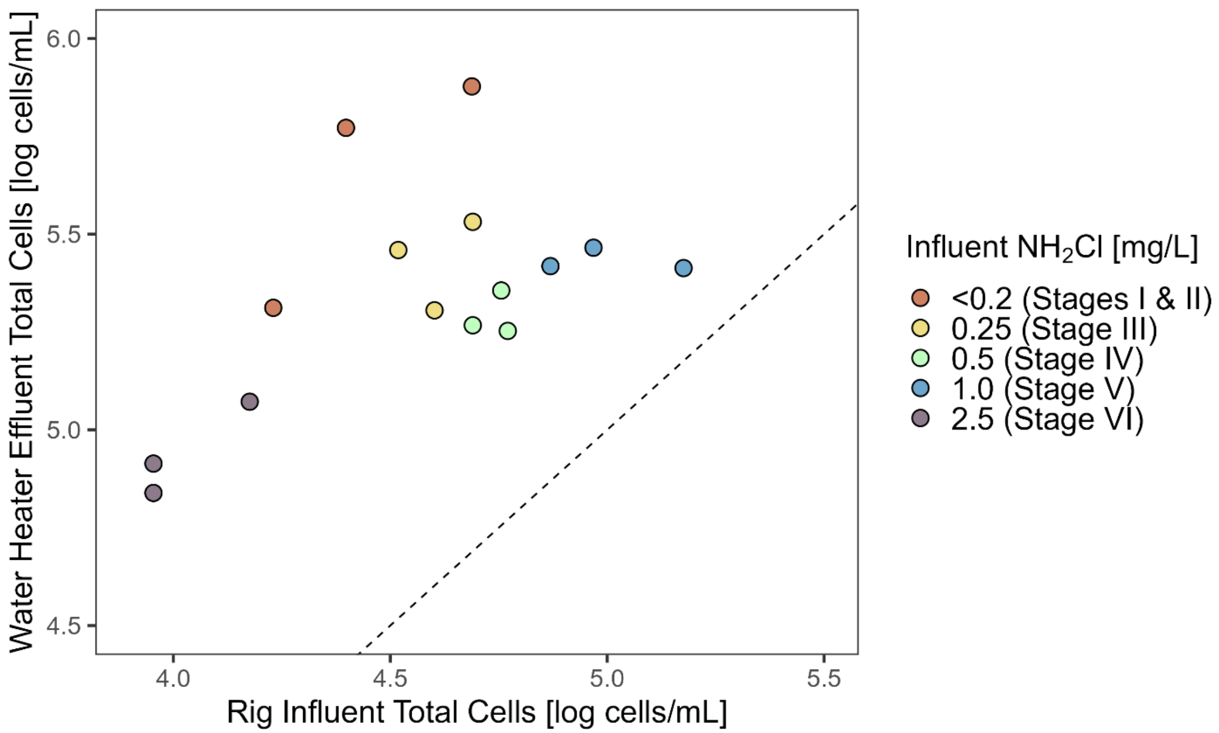

Figure S5. Comparison of total cells in water at the rig influent and water heater effluent. Relationship between total cell count (TCC) at the rig influent and water heater effluent across all stages of influent chloramine. Dashed line represents a positive 1:1 ratio.

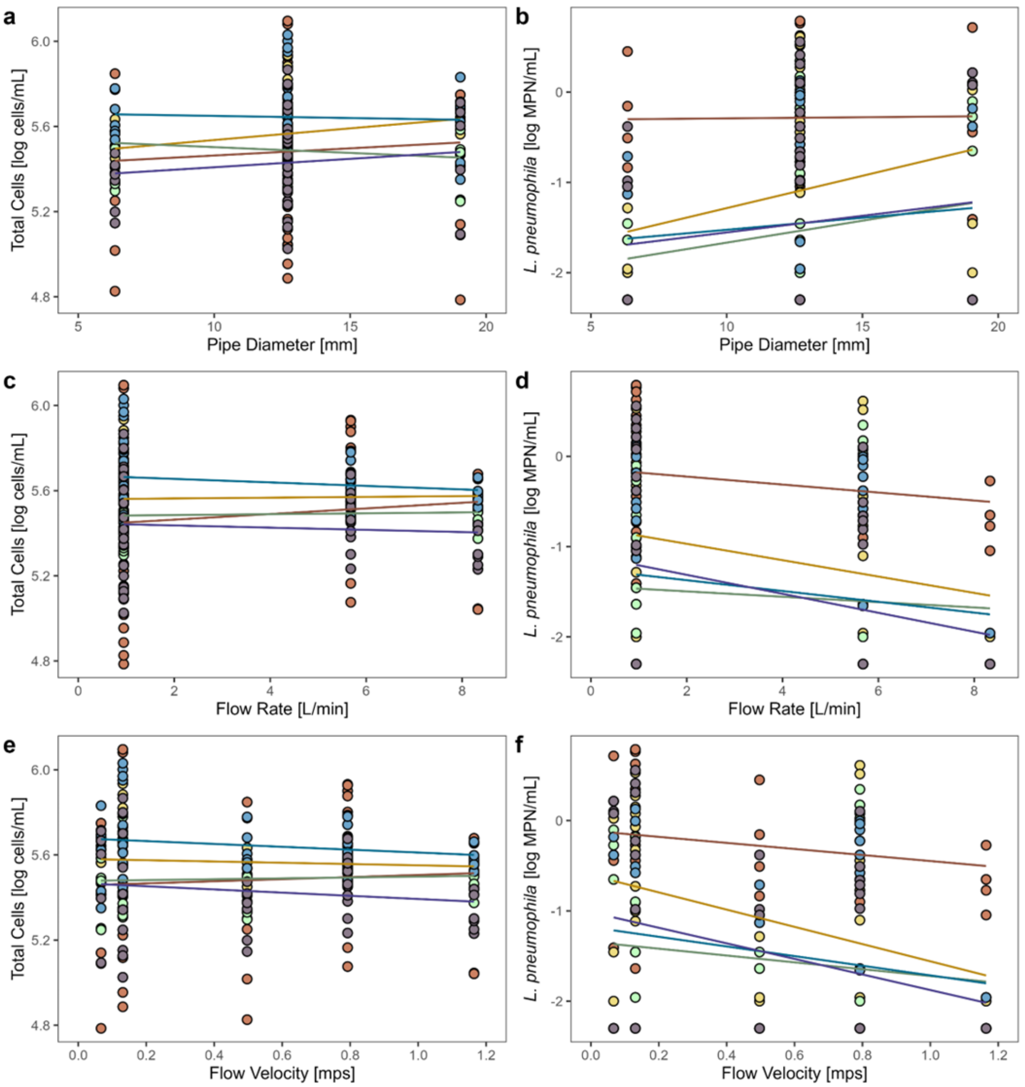

Influent NH_2Cl [mg/L]	<0.2 (Stages I & II)	0.25 (Stage III)	0.5 (Stage IV)	1.0 (Stage V)	2.5 (Stage VI)
a Pipe Diameter (TCC)					
b Pipe Diameter (*Lp*)					
c Flow Rate (TCC)					
d Flow Rate (*Lp*)					
e Flow Velocity (TCC)					
f Flow Velocity (*Lp*)		*			*

Figure S5. Impact of Hydraulic Design Characteristics in Cold Water Pipes. Trends for total cell counts (TCC) and *Legionella pneumophila* (*Lp*) in cold water pipes as a function of pipe diameter (a and b), flow rate (c and d), and flow velocity (e and f). Linear model regression test results under the legend show significance by stage (n=42) with p-value significance thresholds of 0 to <0.001 '***', 0.001 to <0.01 '**', 0.01 to 0.05 '*', and >0.05 ' '.

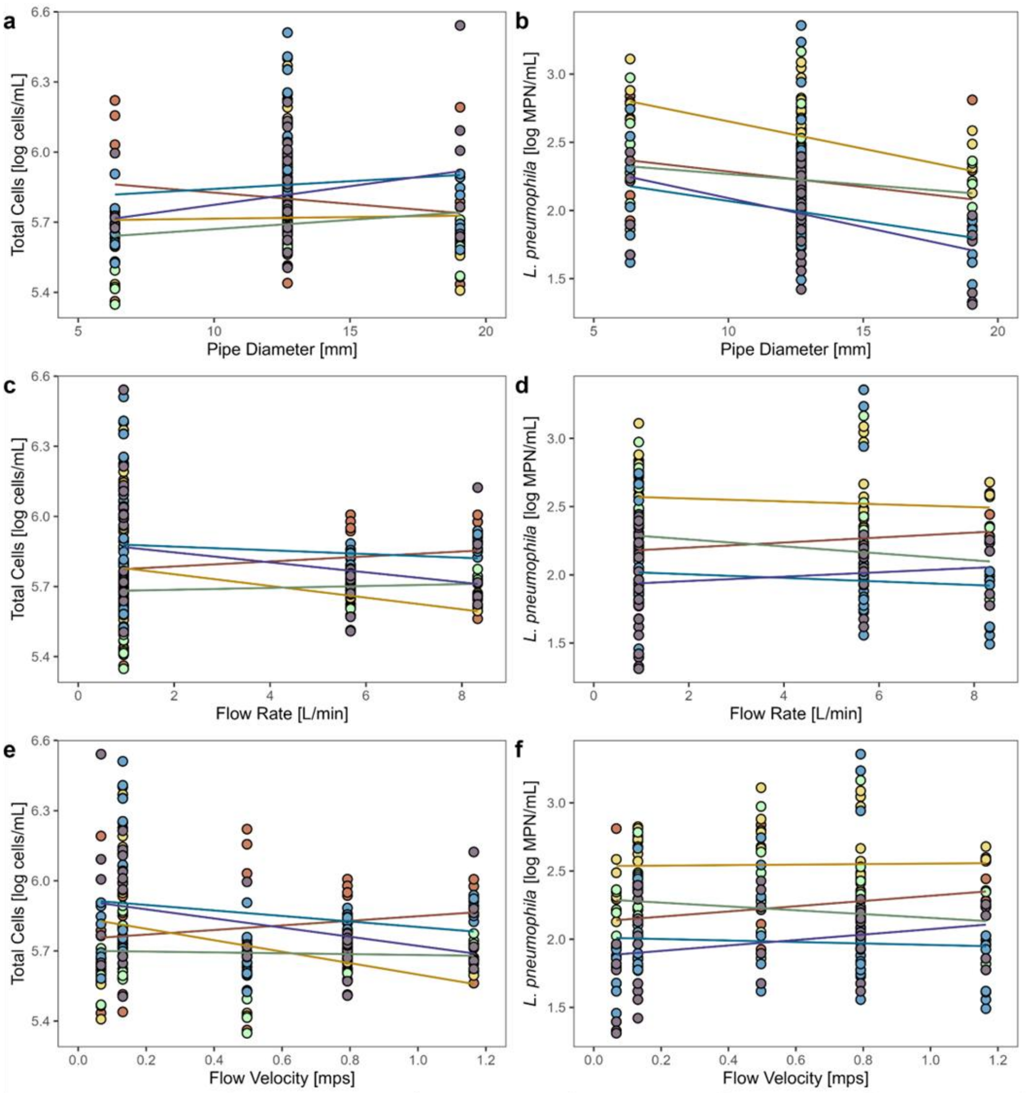

Influent NH_2Cl [mg/L]	<0.2 (Stages I & II)	0.25 (Stage III)	0.5 (Stage IV)	1.0 (Stage V)	2.5 (Stage VI)
a Pipe Diameter (TCC)					
b Pipe Diameter (*Lp*)		**		*	**
c Flow Rate (TCC)					
d Flow Rate (*Lp*)					
e Flow Velocity (TCC)		*			*
f Flow Velocity (*Lp*)		*			*

Figure S6. Impact of Hydraulic Design Characteristics in Hot Water Pipes. Trends for total cell counts (TCC) and *Legionella pneumophila* (*Lp*) in hot water pipes as a function of pipe diameter (a and b), flow rate (c and d), and flow velocity (e and f). Linear model regression test results under the legend show significance by stage (n=42) with p-value significance thresholds of 0 to <0.001 '***', 0.001 to <0.01 '**', 0.01 to 0.05 '*', and >0.05 ' '.

Supplementary Information References

(1) Odimayomi, T. O.; Snead, D. C.; Pruden, A.; Edwards, M. A. Complexity of Chloramine Decay Kinetics in Premise Plumbing. *ACS ES&T Water* **2026**. https://doi.org/10.1021/acsestwater.5c01339.

(2) Nazarian, E. J.; Bopp, D. J.; Saylors, A.; Limberger, R. J.; Musser, K. A. Design and Implementation of a Protocol for the Detection of *Legionella* in Clinical and Environmental Samples. *Diagn. Microbiol. Infect. Dis.* **2008**, *62* (2), 125–132. https://doi.org/10.1016/j.diagmicrobio.2008.05.004.
